# Genomic instability is associated with response to [¹⁷⁷Lu]Lu-PSMA-I&T radioligand therapy: an exploratory, preliminary liquid biopsy analysis

**DOI:** 10.1007/s00259-025-07280-5

**Published:** 2025-04-22

**Authors:** Kilian Kluge, David Haberl, Alexander Haug, Lukas Kenner, Gero Kramer, Shahrokh Shariat, Katarina Kumpf, Marcus Hacker

**Affiliations:** 1 Department of Biomedical Imaging and Image-Guided Therapy, Division of Nuclear Medicine, Medical University of Vienna, Währinger Gürtel 18-20, Vienna, 1090 Austria; 2 Christian Doppler Laboratory for Applied Metabolomics (CDL AM), Medical University of Vienna, Vienna, Austria; 3 Department of Pathology, Medical University of Vienna, Vienna, Austria; 4 Department of Urology, Medical University of Vienna, Vienna, Austria; 5 Department of Urology and Andrology, University Hospital Krems, Krems, Austria; 6 Karl Landsteiner Institute of Urology and Andrology, Vienna, Austria; 7 Department of Urology, University of Texas Southwestern Medical Center, Dallas, USA; 8 Division of Urology, Department of Special Surgery, The University of Jordan, Amman, Jordan; 9 Department of Urology, Second Faculty of Medicine, Charles University, Prague, Czech Republic; 10 Department of Urology, Weill Cornell Medical College, New York, USA; 11 IT Service and Strategic Information Management, Medical University Vienna, Vienna, Austria

**Keywords:** Liquid biopsy, CNV, Prostate cancer, CtDNA, PSMA RLT

## Abstract

**Background:**

PSMA-targeted radioligand therapies (PSMA RLT) are an effective and safe option for metastatic castration-resistant prostate cancer, but responsive subtypes and their biomarkers are not fully defined.

**Methods:**

Plasma samples for cell-free DNA (cfDNA) analysis were collected from 17 patients undergoing [¹⁷⁷Lu]Lu-PSMA-I&T. CfDNA underwent whole-genome sequencing to establish copy number variation (CNV) profiles and circulating-tumor DNA (ctDNA) levels and compared between prostate-specific antigen (PSA) response- and 1-year overall survival (1YOS) groups.

**Results:**

Non-responders exhibited higher degrees of cfDNA CNV burden (*P* = 0.048) and higher ctDNA levels (*P =* 0.036) than responders. Both markers allowed for the differentiation of responses (AUC: 0.792, 0.806) and 1YOS (AUC: 0.778, 0.847).

**Conclusion:**

Unresponsive patients exhibited higher levels of cfDNA genomic instability and ctDNA levels, warranting genome-wide CNV profiling studies next to targeted approaches for mechanistic radiobiological insights and their value as response biomarkers for PSMA RLTs.

**Graphical Abstract:**

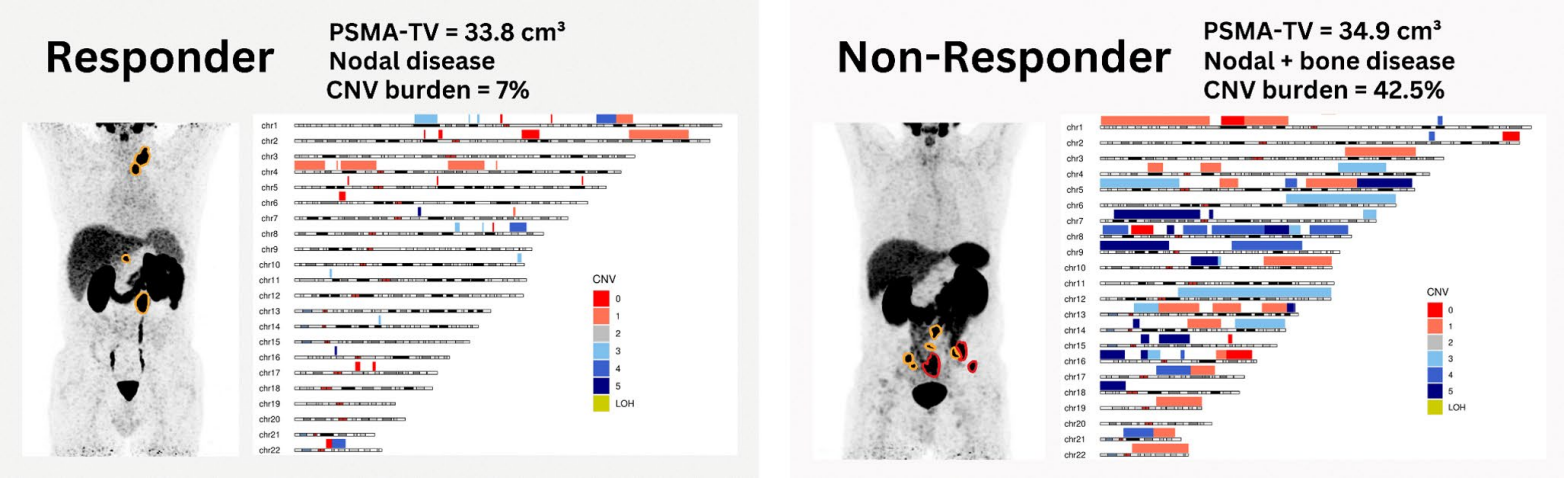

## Introduction

PSMA-targeted radioligand therapies (PSMA RLT), such as the approved [¹⁷⁷Lu]Lu-PSMA-617 [1] or the widely used, non-approved [¹⁷⁷Lu]Lu-PSMA-I&T, are therapeutic options for patients with metastatic castration-resistant prostate cancer (mCRPC).

Target expression probed by PSMA PET is widely appreciated to be predictive of therapeutic success [1–3], while FDG PET [2] and clinical variables, such as disease load, bone and liver metastases, baseline haemoglobin levels and chemotherapy status, have been shown to refine PSMA imaging-based eligibility assessments [3].

However, given the high cost of PSMA RLT and its move to earlier lines of treatment, resulting in more alternative therapeutic options at any given time [4], there is a need for more precise selection methods. In this context, circulating-tumor DNA (ctDNA) analysis has emerged as a promising minimally-invasive source of predictive molecular biomarkers. To date, several reports have linked PSMA RLT responses and outcomes to various somatic aberrations, such as tumor suppressor [5–7], DNA repair [7, 8], cell cycle regulating [7, 9, 10] as well as androgen receptor and PI3K-pathway genes [6, 11] using panel-based ctDNA sequencing approaches. However, genome-wide copy number variations (CNV), structural aberrations characteristic of advanced prostate cancer (PCa) [12–14] and reflective of genomic instability, are underexplored in the context of PSMA RLT, despite their demonstrated value as predictors of taxane chemotherapy responses and outcomes [15, 16]. This pilot aimed to explore this association in the context of PSMA RLT.

## Materials & methods

Patients who underwent plasma sampling at baseline or intermediate [⁶⁸Ga]Ga-PSMA-11 PET/CT staging (Mean 181.6 MBq (± 11.1 SD)) in the context of [¹⁷⁷Lu]Lu-PSMA-I&T RLT were included (03/2019 to 08/2021). All patients underwent at least two cycles of ~ 7.4 GBq of [¹⁷⁷Lu]Lu-PSMA-I&T RLT 6–8 weeks apart. Prostate-specific antigen (PSA) declines at any point after treatment initiation/continuation after sampling were considered. PSA responses were classified naively, with a decline from baseline after therapy considered a response.

The ctDNA and imaging analyses has been described previously [17]. In brief, plasma cfDNA was extracted and quantified using commercially available kits. Next, sequencing libraries were prepared, followed by low-pass whole-genome sequencing (lpWGS). CNV profiles were determined and used to calculate ctDNA fractions analogous to the ichorCNA methodology [18]. The percentage of the genome affected by CNVs was defined as CNV burden [19]. PSMA PET/CT scans were acquired one hour after injection of [⁶⁸Ga]Ga-PSMA-11. PSMA-positive tumour volumes (PSMA-TV) were calculated at both regional and global level, with the dominant tumour fraction defined as the region contributing most to the PSMA-TV.

Numeric variables are reported as mean (± SD) or median (IQR), and discrete outcomes as frequencies (%). Group comparisons used t-tests or Mann-Whitney U test, while discrete outcomes were analysed with chi-squared or Fisher’s exact test, depending on the underlying distributions. Correlations using Spearman’s coefficient after assessing Normality with the Shapiro-Wilk test. Plasma DNA’s predictive potential was evaluated using ROC curve analysis. Overall survival (OS) was analysed using Kaplan-Meier estimates and the Logrank test. All tests assumed a 5% alpha risk.

All patients gave written informed consent for the blood sample collection and associated analysis. This study was approved by the institutional review board of Medical University Vienna, which waived the need for consent for the retrospective part of the analysis (ID: 1649/2016).

## Result

### Clinical cohort

In total, 17 men (age 70.82 ± 7.16 years, PSA 540.76 ± 894.30 ng/dL) with mCRPC who underwent [¹⁷⁷Lu]Lu-PSMA I&T RLT after blood sampling and [⁶⁸Ga]Ga-PSMA-11 PET/CT staging were analysed. The demographic and clinical characteristics are summarised in Table 1.

**Table 1:** Clinical and demographic data before PSMA RLT

Variable	Responder (*N* = 9)	Non-responder (*N* = 8)	*P*
**Age at inclusion [y]**	70.78 (± 7.82) Range: (58.0; 83.0)	70.88 (± 7.38) Range: (57.0; 80.0)	0.979
**cfDNA [ng/µL]**	0.45 (IQR 0.37) Range: (0.269; 2.14)	1.01 (IQR 2.92) Range: (0.247; 9.49)	0.139
**CNV burden [%]**	13.6 (IQR 8.7) Range: (7; 46.1)	24.6 (IQR 16.5) Range: (11.1; 69.3)	**0.048**
**ctDNA detected**	5 (55.56%)	7 (87.5%)	0.294
**ctDNA [ng/µL]**	0.0098 (IQR 0.014) Range: (0.0; 0.296)	0.096 (IQR 0.4) Range: (0.0; 3.12)	**0.036**
**PSA [ng/dL]**	63.8 (IQR 206.96) Range: (0.68; 2800.0)	174.0 (IQR 704.73) Range: (4.15; 1893.0)	0.236
**Hb [g/dL]**	12.08 (± 1.69) Range: (9.4; 14.6)	11.58 (± 1.38) Range: (10.1; 13.5)	0.7
**PSMA-TV [cm³]**	211.15 (IQR 821.95) Range: (0.697; 1439.07)	71.68 (IQR 223.95) Range: (12.04; 863.05)	> 0.999
**PSMA positive lesion**			
Prostate lesion	3 (33.33%)	0 (0.0%)	0.206
Lymph node lesion	7 (77.78%)	5 (62.5%)	0.62
Bone Lesion	6 (66.67%)	7 (87.5%)	0.576
Organ Lesion	2 (22.22%)*†*	1 (12.5%)‡	> 0.999
**Dominant fraction**			> 0.999
Lymph node	4 (44.44%)	3 (37.5%)	
Bone	5 (55.56%)	5 (62.5%)	
**Systemic therapies prior PSMA RLT**			
ADT	6 (66.67%)	4 (50.0%)	0.637
ARPI	4 (44.44%)	7 (87.5%)	0.131
Docetaxel	7 (77.78%)	7 (87.5%)	> 0.999
Cabacitaxel	3 (33.33%)	6 (75.0%)	0.153
PSMA RLT	4 (44.44%)	3 (37.5%)	> 0.999
**Mean Follow-up [m]**	28.11 (± 21.86)	9.89 (± 5.56)	0.052
**1-year OS**			**0.003**
yes	8 (88.89%)	1 (12.5%)	
no	1 (11.11%)	7 (87.5%)	

Qualitative data as numbers and percentages; normally distributed data as mean ± SD, non-normally distributed data as median and interquartile range (IQR); † one patient with an adrenal and liver metastasis, respectively; ‡ patient presented with adrenal and pleural metastasis. Abbreviations: ADT - androgen deprivation therapy, ARPI - androgen receptor pathway inhibitor, Hb– hemoglobin

### ctDNA PSMA-TV correlation

No significant relationship between ctDNA levels (ρ = 0.29; r2 = 0.078; *P* = 0.258) and CNV burden (ρ = 0.1; r2 = 0.017; *P* = 0.701) and PSMA-TV [cm³] as found.

### Response group analysis

**Fig. 1:**
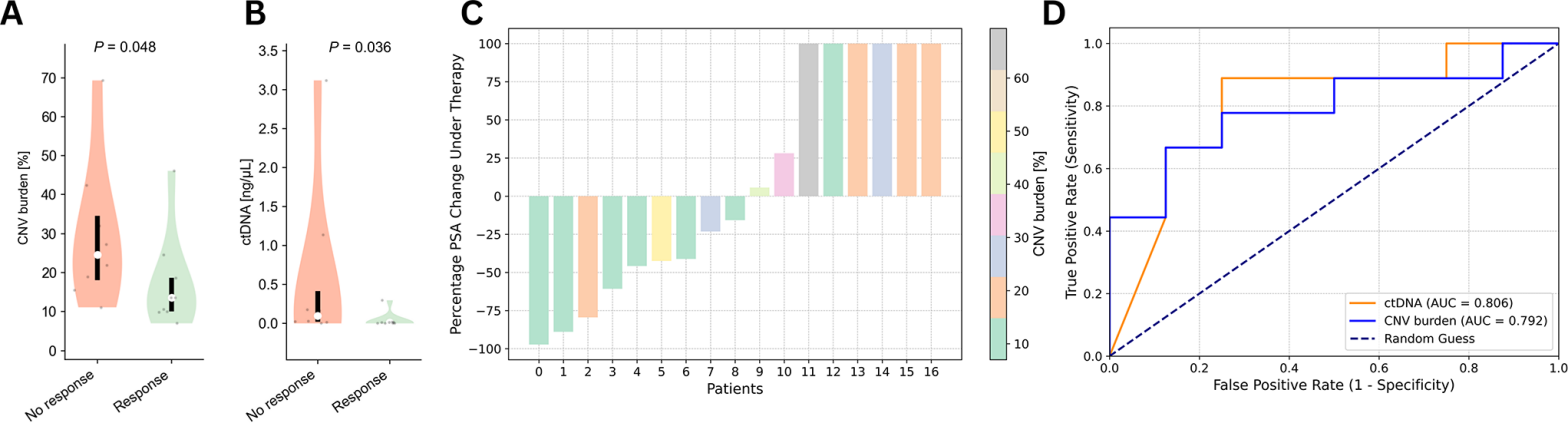
Plasma DNA findings and PSA response **A**) ctDNA levels according to PSA response groups, **B**) cfDNA CNV burden according to PSA response groups, **C**) Waterfall plots of PSA responses according to cfDNA CNV burden, **D**) ROC curves of ctDNA levels and cfDNA CNV burden to predict PSA response

The percentage of cfDNA genome length altered by CNV (CNV burden) differed significantly between responders and non-responders (Median CNV burden 13.6% (IQR 8.7) vs. 24.6 (IQR 16.5); Median Δ= -11.0; *P* = 0.048) (Fig. 1A). Similarly, ctDNA levels [ng/µL] differed significantly between responders and non-responders (Median ctDNA 0.0098 (IQR 0.014) vs. 0.096 (IQR 0.4); Median Δ = -0.087; *P* = 0.036) (Fig. 1B).

Both CNV burden (AUC = 0.792 (95% CI: [0.56; 1.0])) and ctDNA levels (AUC = 0.806 (95% CI: [0.58; 1.0])) differentiated well between responders and non-responders in a ROC analysis (Fig. 1D).

### Survival group analysis

**Fig. 2:**
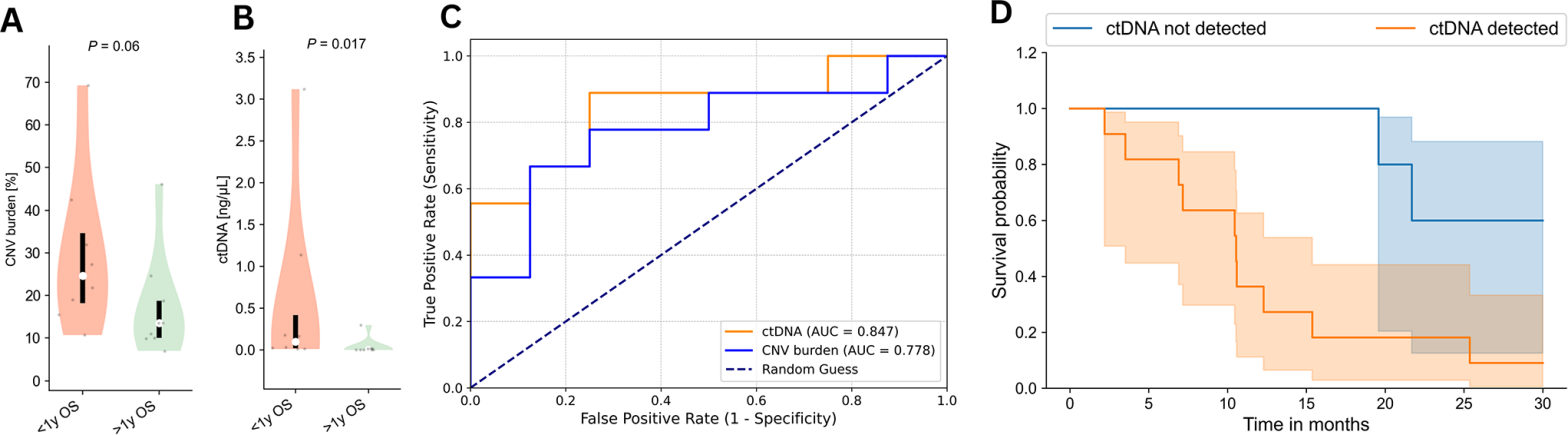
Plasma DNA findings and survival **A**) ctDNA levels according to 1YOS groups, **B**) cfDNA CNV burden according to 1YOS groups, **C**) ROC curves of ctDNA levels and cfDNA CNV burden to predict 1YOS, **D**) Kaplan-Meier curves according to CNV ctDNA detection

Similarly, CNV burden trended higher in patients with an OS shorter than one year compared with patients with an OS longer than one year (Median CNV burden 13.6% (IQR 8.7) vs. 24.6 (IQR 16.5); Median Δ= − 11.0; *P* = 0.06) (Fig. 2A). Conversely, ctDNA levels [ng/µL] differed significantly between patients with an OS shorter and longer than one year (Median ctDNA 0.0 (IQR 0.014) vs. 0.096 (IQR 0.4); Median Δ = -0.097; *P* = 0.017) (Fig. 2B).

Analogously to the response differentiation, both CNV burden (AUC = 0.778 (95% CI: [0.54; 1.0])) and ctDNA levels (AUC = 0.847 (95% CI: [0.65; 1.0])) differentiated well between patients according to 1YOS (Fig. 2C).

There was a difference in survival of patients with and without detectable ctDNA (*P* = 0.019) (Fig. 2D).

## Discussion

Patients who were PSA non-responders and those with OS of less than one year exhibited significantly higher cfDNA CNV burden and CNV-based ctDNA levels.

CNVs are structural genomic variations constituted mainly by amplifications and deletions [20], which can accumulate as DNA repair and cell cycle control mechanisms fail [20, 21]. These findings align with previous investigations of PSMA RLT responsive genotypes using tumor samples or liquid biopsies.

For example, Kratochwil et al. reported that lesions non-responsive to Actinium-225 PSMA-617 RLT frequently harboured mutations in DNA damage repair (DDR) and cell cycle controlling/tumor suppressor genes (TSG) [22]. Similarly, Crumbaker et al. [5], De Bono et al. [6] and preliminary results by Fettke et al. [8] reported TSGs and cell cycle controlling gene defects to be negatively associated with PSMA responses. Interestingly, Raychaudhuri et al. [7] reported that DDR-mutated, particularly ATM harbouring, and mismatch-repair deficient PCas were more likely to respond to PSMA RLT than TSG-mutated PCas.

Against the backdrop of these previous reports [5–8], we hypothesize that the observed elevated CNV burden and CNV-based ctDNA levels in non-responders may result from a combination of defective DNA repair and impaired cell cycle control leading to a tolerance to progressive accumulation of genomic aberrations without triggering apoptosis. Conversely, in patients with intact cell cycle control mechanisms but defective DNA repair machinery [7], non-repairable DNA damage induced by RLTs might trigger apoptosis in the presence of a functioning cell cycle regulation, thereby conveying radiosensitivity.

Irrespective of ontogenic mechanistic speculations, our findings are further empirically corroborated by reports of Sartor et al. [9], who found PSA non-responders to harbour significantly more amplifications using a commercial, targeted 83-gene panel assay, and speculated that this might reflect genomic instability at larger scale. Furthermore, Sumanasuriya et al. [15] found that lpWGS CNV-based ctDNA analysis predicted taxane chemotherapy response and outcome [15, 16] and that genomic instability was associated with prior abiraterone and enzalutamide therapy, but not with prior taxane- or radiation therapy. Here our data also tended towards higher androgen receptor pathway inhibitor levels in the non-responder group.

Based on the generated data, we speculate that the CNV-based estimation of ctDNA fraction could potentially be a valuable complementary PSMA RLT selection strategy, providing a high-level, cost-effective aggregate overview of the genomic integrity [23].

There are several limitations to this preliminary analysis, for which the results should be interpreted with caution. First, it was naturally limited by its small monocentric nature including a heterogeneous treatment population at various timepoints during the PSMA RLT treatment. This limits the statistical robustness of the conclusions drawn, particularly as the small sample size did not allow for a multivariate analysis to exclude potential confounders. Second, the relationship between underlying single nucleotide polymorphisms and insertion/deletion with CNV profiles was not possible due to lacking data, which hinders mechanistic interpretations. This issue should be addressed in comprehensive, more homogeneous cohorts using integrated low-pass WGS and high-coverage targeted sequencing approaches in the future.

Despite these limitations, our findings align closely with previous reports and thereby strongly corroborate earlier speculations regarding the association between genetic instability and PSMA RLT outcomes. This warrants further investigations into genome-wide ctDNA CNVs profiles alongside currently employed panel-based sequencing approaches for mechanistic radiobiological insights and their potential value as response biomarkers for PSMA RLTs.

## Conclusion

Patients unresponsive to PSMA RLT exhibited higher levels of plasma DNA genomic instability and ctDNA levels, which warrants investigations into genome-wide ctDNA CNVs profiles next to currently employed panel-based sequencing approaches for mechanistic radiobiological insights and their value as response biomarkers for PSMA RLTs.
